# Can Inconsistent Association between Hypertension and Cognition in Elders be Explained by Levels of Organochlorine Pesticides?

**DOI:** 10.1371/journal.pone.0144205

**Published:** 2015-12-02

**Authors:** Se-A Kim, Yu-Mi Lee, Ho-Won Lee, David R. Jacobs, Duk-Hee Lee

**Affiliations:** 1 Department of Biomedical Science, Kyungpook National University, Daegu, Korea; 2 BK21 Plus KNU Biomedical Convergence Program, Department of Biomedical Science, Kyungpook National University, Daegu, Korea; 3 Department of Preventive Medicine, School of Medicine, Kyungpook National University, Daegu, Korea; 4 Department of Neurology, School of Medicine, Kyungpook National University, Daegu, Korea; 5 Brain Science & Engineering Institute, Kyungpook National University, Daegu, Korea; 6 Division of Epidemiology, School of Public Health, University of Minnesota, Minneapolis, Minnesota, USA; Institute for Health & the Environment, UNITED STATES

## Abstract

The relation between hypertension and cognition in elders remains unclear, and studies on the effect of antihypertensive drugs on cognition have demonstrated conflicting results. This study was performed to evaluate if the association between hypertension and cognition in elders differed depending on serum concentrations of organochlorine (OC) pesticides, common neurotoxic chemicals. Participants were 644 elders aged 60–85 years who participated in the National Health and Nutrition Examination Survey 1999–2002 and were able to complete a cognitive test. We selected 6 OC pesticides that were commonly detected in the elderly. Cognition was assessed by the Digit Symbol Substitution Test (DSST), a relevant tool for evaluating hypertension-related cognitive function, and low cognition was defined by the DSST score < 25^th^ percentile. When OC pesticides were not considered in the analyses, elders with hypertension had about 1.7 times higher risk of low cognition than those without hypertension. However, in analyses stratified by serum concentrations of OC pesticides, the associations between hypertension and low cognition were stronger the higher the serum concentrations of p,p’-DDT, p,p’-DDE, β-hexachlorocyclohexane, and trans-nonachlor increased. Among elders in the 3^rd^ tertile of these pesticides, adjusted odds ratios were from 2.5 to 3.5. In contrast, hypertension was not clearly associated with the risk of low cognition in elders in the 1^st^ tertile of these pesticides. Similar patterns were observed for the continuous DSST score dependent variable. The difference in the association between hypertension and DSST scores according to the levels of OC pesticides suggest a key role of OC pesticides in the development of hypertension-related cognitive impairment and may help to identify hypertensive elders who are at a high risk of cognitive impairment.

## Introduction

High blood pressure in midlife is associated with an increased risk of dementia, including Alzheimer's disease and vascular dementia, through accelerating arteriosclerotic changes in the brain [1]. However, the relationship of high blood pressure later in life with cognition remains unclear [1]. Randomized controlled studies have demonstrated heterogeneous effects of blood pressure lowering on cognitive function [2]. At present, it is unknown whether there is any modifying effect of other variables on the relationship of blood pressure with cognition which may be helpful in interpreting inconsistent results in elders.

Recently, background exposure to organochlorine (OC) pesticides, which are strong lipophilic chemicals with long half-lives of several months to years, are stored in adipose tissues, and are continuously released into circulation, has been linked to decreased cognition [3–5] and to Alzheimer's disease in the elderly [3–5]. In particular, some OC pesticides were associated with accelerated aging-related cognitive impairment [6].

Even though high blood pressure has been generally considered as a pathophysiological change, a certain level of blood pressure may be necessary for older adults to maintain cerebral perfusion, and thereby to preserve cognitive ability [1]. In fact, poor cerebral perfusion is suspected to be one of the pathways linking vascular disorders to dementia [7, 8]. However, this possible benefit of blood pressure in elders can be counteracted by the presence of neurotoxic OC pesticides in the blood because high blood pressure may increase the chance that these chemicals in blood reach neuronal cells.

Therefore, this study was performed to investigate if the association between hypertension and cognition in the elderly would differ depending on serum concentrations of OC pesticides, common neurotoxic chemicals. Specifically, we hypothesized that the hypertension association would be stronger at higher serum concentrations of OC pesticides. Among various domains of cognitive function, the Digit Symbol Substitution Test (DSST) was used in this study. DSST primarily evaluates psychomotor speed within a comprehensive assessment of cognitive function [9]. Hypertension is more related to worse performance and more rapid decline in executive function and processing speed than memory performance [10, 11], so that the DSST is a highly relevant tool for evaluating hypertension-related cognitive function.

## Materials and Methods

### Study subjects

The National Health and Nutrition Examination Survey (NHANES) conducted annually since 1999 by the Centers for Disease Control and Prevention (CDC) is an ongoing survey designed to measure the health and nutritional status of the civilian noninstitutionalized U.S. population. Cognitive function was measured in all elders aged 60~85 of the NHANES 1999–2002 who participated in the household interview while serum organochlorine pesticides were measured in the randomly selected subsample of the NHANES 1999–2002. Among 751 elderly who initially participated in cognitive function test and had information on organochlorine pesticides, 107 elderly was excluded because they were unable to complete sample items and did not continue with the remainder of the exercise. Only a small number of subjects provided specific reasons unable to complete sample items (14 for physical limitations; 9 for cognitive limitations, and others). Thus, the final sample size was 644 people who did complete the DSST test. The study protocol was reviewed and approved by the institutional review board of the Centers for Disease Control in the U.S. Also, informed written consent was obtained from all subjects before they took part in the study.

### Measurement

The NHANES standardized home interview was followed by a detailed physical examination in a mobile evaluation clinic or the participant's home. Information about existing medical conditions was collected using questionnaires. Blood pressure of all eligible participants was measured sphygmomanometrically, and at least three readings of systolic and diastolic blood pressures were recorded using the latest recommendations of the American Heart Association.[12] We used the average values of systolic and diastolic blood pressures, which were reported to the participants.

Cognitive function was assessed using DSST. The DSST, a component of the Wechsler Adult Intelligence Test, is a test of visuospatial and motor speed-of-processing, has a considerable executive function component, and is frequently used as a sensitive measure of frontal lobe executive function.[13] The task consisted of drawing unfamiliar symbols under the corresponding number, resulting in a score indicating the number of correct symbols drawn within a period of 120 seconds with a maximum score of 133.

Venous blood samples were collected and shipped weekly at -20°C. OC pesticides were all measured as individual chemicals by high-resolution gas chromatography/high-resolution mass spectrometry using isotope dilution for quantification. We selected 6 organochlorine pesticides (p,p’- dichlorodiphenyltrichloroethane (DDT), p,p’- dichlorodipenyldichloroethylene (DDE), β-hexachlorocyclohexane, trans-nonachlor, oxychlordane, and heptachlor epoxide) which were associated with DSST score in a previous study [3]. For lipid-standardized concentrations, each organochlorine concentration was divided by the total lipid value (Total lipids (mg/dl) = 2.27 x total cholesterol+triglycerides+62.3) [14]. Results of lipid-standardized concentrations were similar to those of wet concentrations adjusted for lipids in statistical models [3]. We presented results based on lipid-standardized concentrations.

### Statistical analysis

Participants were considered to have hypertension if they were treated with antihypertensive drugs or if their systolic or diastolic blood pressure was ≥140 mmHg or ≥90 mmHg. In this study, we used both continuous DSST score and dichotomous DSST score < 25% (DSST score: 28) as outcome variables. General linear models and logistic regression models were used to calculate adjusted means and multivariate-adjusted odd ratios (ORs).

First, we evaluated associations of hypertension with DSST score and the risk of low cognitive function among all subjects. Next, we performed stratified analyses by tertiles of serum concentrations of OC pesticides which were defined following the distribution of all study subjects. In addition to stratified analyses, we presented adjusted ORs when elders without hypertension within the 1^st^ tertile of OC pesticides were used as the common reference group.

Covariates were age (continuous), gender, race-ethnicity (non-Hispanic white, non-Hispanic black, Mexican American, multiracial, or others), education (less than 9^th^ grade, 9^th^-11^th^ grade, and high school graduates), poverty income ratio (the ratio of self-reported family income to the family's appropriate threshold value, continuous), smoking status (current, ex, and never), and body mass index (continuous). We further considered history of physician-diagnosed heart diseases, diabetes, and cancer as possible covariates.

Estimates of main results were calculated accounting for NHANES stratification and clustering [15] and were adjusted for age, gender, and race-ethnicity instead of using sample weights; this adjustment has been regarded as a good compromise between efficiency and bias [15, 16]. We present the results based on SAS 9.3.

## Results

The characteristics of our study subjects by the status of hypertension are presented in Table 1. Subjects with hypertension were older and more likely to be women than those without hypertension. The mean score of DSST was 44.5 among elders without hypertension while it was 39.1 among those with hypertension.

**Table 1:** Characteristics of study subjects.

Characteristics	Hypertension		
	No (n = 207)	Yes (n = 437)	P value*
Mean ± standard deviation (range)			
Age, years	69.8±7.6 (60–85)	71.8±7.8 (60–85)	<0.01
Poverty income ratio	2.5±1.3 (0–5)	2.3±1.4 (0–5)	0.07
Body mass index, kg/m^2^	27.9±5.7 (16.2–49.5)	28.6±5.3 (16.4–49.7)	0.17
Score of Digit Symbol Substitution Test	44.5±18.4 (1–89)	39.1±18.1 (0–113)	<0.01
Number of subjects (%)			
Men	114 (55.1%)	189 (43.3%)	<0.01
White	125 (60.4%)	258 (59.0%)	0.74
High school graduate	71 (34.3%)	148 (33.9%)	0.69
Current smoker	25 (12.1%)	46 (10.5%)	0.56
Body mass index ≥ 30 kg/m^2^	57 (27.5%)	136 (31.1%)	0.35
Physician-diagnosed heart diseases	25 (12.1%)	97 (22.2%)	<0.01
Physician-diagnosed diabetes	23 (11.1%)	89 (20.4%)	<0.01
Physician-diagnosed cancer	33 (15.9%)	93 (21.3%)	0.11

*P values were calculated by Student T-test for continuous variables or X^2^ test for categorical variables.

Table 2 shows associations between hypertension and DSST score among all subjects and stratified by tertiles of serum concentrations of OC pesticides. When OC pesticides were not considered in the analyses, elders without hypertension had a higher score by about 3.8 points than those with hypertension (P < 0.01). This association was not materially different after further adjustment for history of physician-diagnosed heart diseases, diabetes, and cancer (S1 Table). However, when we performed stratified analyses by serum concentrations of OC pesticides, the association between hypertension and DSST score generally became stronger as serum concentrations increased. In particular, when elders had the 1st tertile of p,p’-DDT, p,p’-DDE, β-hexachlorocyclohexane, and trans-nonachlor, the DSST scores of elders with and without hypertension did not differ significantly. P values for interaction for p,p’-DDT and p,p’-DDE were 0.06 and 0.01, respectively while β-hexachlorocyclohexane and trans-nonachlor did not reach the statistical significant level despite a similar pattern.

**Table 2:** Adjusted* means of Digit Symbol Substitution Test score among all subjects or stratified by tertiles of serum concentrations of organochlorine pesticides.

		Hypertension (-) (n = 207)	Hypertension (+) (n = 437)	P _value_	P_interaction_
All subjects		43.4	39.6	<0.01	
Stratified analyses by tertiles of each compound (median value, interquartile ranges, unit: ng/g lipid)					
p,p’-DDT	T1 (5.7, 5.0~6.2)	46.0	45.0	0.62	0.06
	T2 (9.4, 8.1~11.3)	45.3	41.2	0.07	
	T3 (25.6, 18.2~48.7)	39.1	33.1	<0.01	
p,p’-DDE	T1 (324.5, 216~470)	44.1	44.0	0.97	0.01
	T2 (940.5, 751~1170)	44.6	39.9	0.04	
	T3 (2200, 1660~3240)	42.4	35.0	<0.01	
β-hexachlorocyclohexane	T1 (12.8, 9.2~16.8)	44.7	42.1	0.22	0.39
	T2 (28.5, 23.0~35.2)	45.2	41.0	0.05	
	T3 (73.4, 53.7~114.0)	40.5	36.0	0.05	
Trans-nonachlor	T1 (25.9, 20.7~31.5)	45.1	43.0	0.36	0.14
	T2 (48.2, 41.7~53.4)	45.5	41.4	0.05	
	T3 (88.9, 71.7~133.0)	40.1	34.4	0.01	
Oxychlordane	T1 (17.9, 14.3~21.1)	45.3	41.5	0.09	0.62
	T2 (31.3, 28.1~35.2)	45.9	42.6	0.11	
	T3 (54.7, 46.0~73.9)	39.1	34.8	0.06	
Heptachlor epoxide	T1 (5.1, 3.9~6.1)	45.9	42.1	0.10	0.66
	T2 (10.7, 9.2~12.3)	44.5	40.5	0.03	
	T3 (23.0, 17.5~34.6)	38.6	36.6	0.42	

*Adjusted for age, sex, race-ethnicity, education, poverty income ratio, cigarette smoking, and body mass index.

When we used the risk of low cognition as the outcome (Table 3, S2 Table), the results were similar to those obtained using the continuous DSST score. Among all subjects, elders with hypertension had about 1.7 times higher risk of low cognition than those without hypertension. In the analyses stratified by serum concentrations of OC pesticides, again when elders had p,p’-DDT, p,p’-DDE, β-hexachlorocyclohexane, and trans-nonachlor in the 1^st^ tertile, hypertension was not clearly associated with the risk of low cognition. However, for elders with these OC pesticides in the 3^rd^ tertile, hypertension was associated with a 2.5–3.5 fold higher odds ratios for low cognitive score.

**Table 3:** Adjusted* odds ratios (ORs) and 95% confidence intervals (CIs) between hypertension and the risk of low Digit Symbol Substitution Test score (<25^th%^ of study subjects) among all subjects or stratified by serum concentrations of organochlorine (OC) pesticides.

		Hypertension (-) (n = 207)	Hypertension (+) (n = 437)	P _value_	P_interaction_
All subjects	Cases/Subjects at risk	40/207	128/437		
	Adjusted ORs (95% CIs)	Reference	1.7 (1.1–2.7)		
Stratified analyses by tertiles of each compound					
p,p’-DDT					
T1	Cases/Subjects at risk	11/87	23/126		
	Adjusted ORs (95% CIs)	Reference	1.6 (0.7–3.9)	0.28	0.46
T2	Cases/Subjects at risk	12/62	40/156		
	Adjusted ORs (95% CIs)	Reference	1.2 (0.5–3.0)	0.63	
T3	Cases/Subjects at risk	17/58	65/155		
	Adjusted ORs (95% CIs)	Reference	2.5 (1.1–5.5)	0.03	
p,p’-DDE		Hypertension(-)	Hypertension (+)		
T1	Cases/Subjects at risk	15/76	27/138		
	Adjusted ORs (95% CIs)	Reference	0.8 (0.4–1.9)	0.68	0.05
T2	Cases/Subjects at risk	12/70	38/144		
	Adjusted ORs (95% CIs)	Reference	2.1 (0.9–5.1)	0.08	
T3	Cases/Subjects at risk	13/61	63/155		
	Adjusted ORs (95% CIs)	Reference	2.8 (1.2–6.5)	0.02	
β-hexachlorocyclohexane		Hypertension(-)	Hypertension (+)		
T1	Cases/Subjects at risk	18/89	27/125		
	Adjusted ORs (95% CIs)	Reference	1.3 (0.6–2.8)	0.48	0.21
T2	Cases/Subjects at risk	11/64	41/151		
	Adjusted ORs (95% CIs)	Reference	1.6 (0.7–3.9)	0.29	
T3	Cases/Subjects at risk	11/54	60/161		
	Adjusted ORs (95% CIs)	Reference	2.4 (1.0–5.7)	0.05	
Trans-nonachlor		Hypertension(-)	Hypertension (+)		
T1	Cases/Subjects at risk	14/77	34/138		
	Adjusted ORs (95% CIs)	Reference	1.2 (0.5–2.9)	0.63	0.06
T2	Cases/Subjects at risk	14/67	33/147		
	Adjusted ORs (95% CIs)	Reference	1.3 (0.6–2.9)	0.53	
T3	Cases/Subjects at risk	12/63	61/152		
	Adjusted ORs (95% CIs)	Reference	3.3 (1.4–8.0)	<0.01	
Oxychlordane		Hypertension(-)	Hypertension (+)		
T1	Cases/Subjects at risk	14/83	41/130		
	Adjusted ORs (95% CIs)	Reference	2.8 (1.2–6.4)	0.02	0.86
T2	Cases/Subjects at risk	13/66	28/150		
	Adjusted ORs (95% CIs)	Reference	0.9 (0.3–1.8)	0.58	
T3	Cases/Subjects at risk	13/58	59/157		
	Adjusted ORs (95% CIs)	Reference	2.5 (1.1–5.7)	0.03	
Heptachlor epoxide		Hypertension(-)	Hypertension (+)		
T1	Cases/Subjects at risk	21/125	20/101		
	Adjusted ORs (95% CIs)	Reference	1.7 (0.8–3.7)	0.20	0.95
T2	Cases/Subjects at risk	10/43	34/142		
	Adjusted ORs (95% CIs)	Reference	1.8 (0.7–4.4)	0.22	
T3	Cases/Subjects at risk	15/53	58/160		
	Adjusted ORs (95% CIs)	Reference	1.5 (0.6–3.3)	0.37	

*Adjusted for age, sex, race-ethnicity, education, poverty income ratio, cigarette smoking, and body mass index.

**Fig 1:**
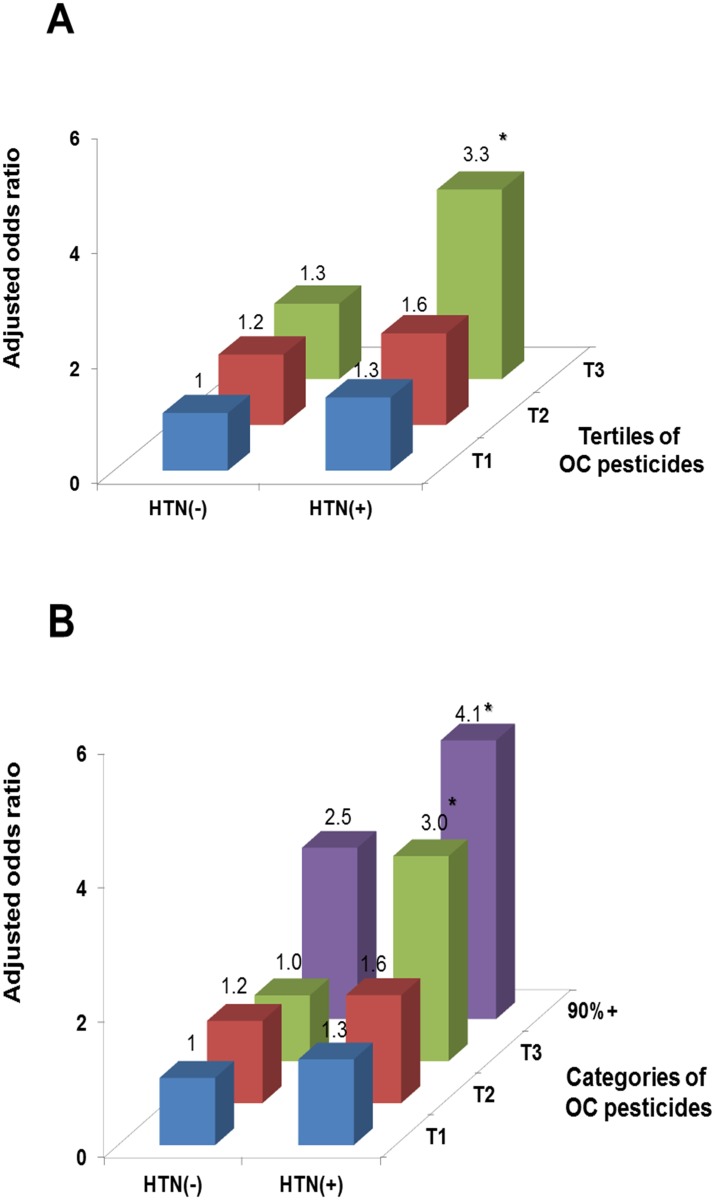
Associations of hypertension (HTN) and summary measure of organochlorine (OC) pesticides with the risk of low cognitive score (<25th % of study subjects, 28 of 133 symbols correctly substituted). The summary measure was calculated by summing the rank orders of the individual 4 compounds (p,p’-DDT, p,p’-DDE, β-hexachlorocyclohexane, and trans-nonachlor) which showed little association in the 1st tertile, but the significant positive association in the 3rd tertile in Table 3. Odds ratios were estimated using a common reference group of elderly without HTN within the 1st tertile of OC pesticides, adjusted for age, sex, race-ethnicity, education, poverty income ratio, cigarette smoking, and body mass index. The upper fig A used tertiles of the summary measure of OC pesticides, while the lower fig B further categorized the last tertile by the cutoff point of 90%. Statistically significant odds ratios were marked with *. T1, first tertile; T2, second tertile; T3, third tertile. Sample sizes are given in Table 3.

Fig 1 shows the adjusted ORs across tertiles of the summary measure of OC pesticides, using the 1^st^ tertile within elders without hypertension as the common reference group. The summary measure was calculated by summing the rank orders of the 4 individual compounds (p,p’-DDT, p,p’-DDE, β-hexachlorocyclohexane, and trans-nonachlor), each of which showed little association between hypertension and low DSST in the 1st tertile, but had significant positive association in the 3^rd^ tertile in Table 3. The ORs for low cognition among elders with hypertension within the 3^rd^ tertile was about 3.3 (95% confidence interval: 1.5–7.5) (Fig 1A), compared to 1.3 within the 1^st^ tertile. From the opposite viewpoint of the summary measure of OC pesticides, only hypertensive elderly seemed to show a positive association between OC pesticides and risk of low cognition. However, when the 3^rd^ tertile was further categorized by splitting the 3^rd^ tertile at the cutoff point of the 90^th^ percentile (Fig 1B), non-hypertensive elderly also showed an apparent positive trend in the last category of OC pesticides. Thus, the risk of low cognition increased in hypertensive elderly at lower doses of OC pesticides, compared to the risk of low cognition among non-hypertensive elderly. Results considering NHANES study designs and sample weights showed similar trends, but statistical significances were weaker (S1 Fig).

## Discussion

This study demonstrated that the association between hypertension and cognitive function measured by the DSST in the elderly differed depending on the serum concentrations of p,p’-DDT, p,p’-DDE, β-hexachlorocyclohexane, and trans-nonachlor. In the stratified analyses, the strength of association between hypertension and low cognitive score increased as serum concentrations of these OC pesticides increased. In contrast, hypertension was not or only weakly associated with the risk of low cognition among elderly with low concentrations of these OC pesticides. These data are consistent with an important role for OC pesticides in accelerating the pathogenesis of hypertension-related cognitive impairment.

It is generally accepted that hypertension accelerates arteriosclerotic changes in the brain, predisposing to atheroma formation in large diameter blood vessels and arteriosclerosis and arteriolar tortuosity of small vessels of the cerebral vasculature [17]. Neuropathological studies have linked arteriosclerotic burden in the brain among hypertensive patients to the pathological changes of both Alzheimer's disease and vascular cognitive impairment [18, 19].

Despite the biological plausibility, however, the relation between blood pressure and cognition in elders remains a subject of considerable controversy [1]. Findings from human studies are diverse from inverse to positive associations between blood pressure and cognition [1]. In addition, intervention studies with anti-hypertensives have shown opposing effects of blood pressure reduction on cognitive function [2]. At present, much of the controversy is attributed to methodological issues such as differences in subjects, assessment methods for cognitive function, and confounding effects [2]. An alternative viewpoint is that high blood pressure among elders might be required to a certain level for adequate perfusion to brain, especially when cerebral arteries are diseased [20].

Interestingly, the current study demonstrated that OC pesticides in blood may be helpful in explaining the inconsistent associations on hypertension and cognition in elderly. As common neurotoxins that move with blood lipids, OC pesticides were recently linked to cognitive impairment and Alzheimer’s disease in the elders [3–5]. As OC pesticides easily cross the blood brain barrier [21], it is reasonable to assume that the chronic presence of these chemicals in blood can influence neurological effects of high blood pressure. Furthermore, various lipophilic chemicals coexisting with OC pesticides as mixtures in human are linked to atherosclerosis [22–24] and stroke [25], which are considered as pathological mechanisms linking hypertension and cognitive impairment. Thus, these lipophilic chemicals in blood can directly and indirectly affect hypertension-related cognitive impairment.

From the 1940s to 1970s, OC pesticides were widely marketed and applied as insecticides for crop protection and for control of vector-borne diseases such as malaria and typhus. Even though they have been banned in most developed countries for over 30 years, they remain in the environment because of their long half-lives. DDT is still permitted to be produced and used for controlling disease vectors of malaria according to recommendations of the World Health Organization[26]. OC pesticides have strong lipophilicity, tend to accumulate in food sources and adipose tissues, and move with lipids in blood. The current elderly population is the first generation with a life-time exposure to OC pesticides.

This study has several limitations. First, the cross-sectional study design precludes the establishment of temporal relationships even though the effects of the interaction between hypertension and OC pesticides on the risk of low cognitive function may be difficult to explain by reverse causality. Second, only the DSST was used to evaluate cognitive function. Cognition is generally assessed through a battery of psychometric tests repeatedly administered to the subjects. Collecting several cognitive tests may be useful, because this allows exploration of the various cognitive domains (memory functioning, attention, or executive functions) and because the tests often have different metric properties. Third, the outcome used in this study was not definite dementia. Even though cognitive function in older people is reportedly a predictor of dementia [27], future studies with clinically relevant outcomes are required.

The present study demonstrated that the association between hypertension and DSST score was modified by the levels of OC pesticides. No or weak association between hypertension and DSST score among elderly with low concentrations of OC pesticides suggests the possibility of a certain role of OC pesticides in the development of hypertension-induced dementia. Also, these findings could help to identify hypertensive elders who are at a high risk of dementia and would benefit most in this respect from antihypertensive therapy for lowering blood pressure.

## Supporting Information

S1 Fig Associations of hypertension (HTN) and summary measure of organochlorine (OC) pesticides with the risk of low cognitive score. Different from Fig 1, adjusted odds ratios were estimated after considering the design variables and sample weight of National Health and Nutrition Examination Survey (NHANES)
(DOCX)

S1 Table Adjusted* means of Digit Symbol Substitution Test score among all subjects or stratified by tertiles of serum concentrations of organochlorine pesticides. (DOCX)

S2 Table Adjusted* odds ratios (ORs) and 95% confidence intervals (CIs) between hypertension and the risk of low Digit Symbol Substitution Test score (<25^th%^ of study subjects) among all subjects or stratified by serum concentrations of organochlorine (OC) pesticides. (DOCX)
