# Supplementary material for: Can Inconsistent Association between Hypertension and Cognition in Elders be Explained by Levels of Organochlorine Pesticides?
Source: PLoS One. 2015 Dec 2;10(12):e0144205. doi: 10.1371/journal.pone.0144205 (PMC4668046; doi:10.1371/journal.pone.0144205)
Supplement: S1 Fig — Different from Fig 1, adjusted odds ratios were estimated after considering the design variables and sample weight of National Health and Nutrition Examination Survey (NHANES) (DOCX) [file pone.0144205.s001.docx]

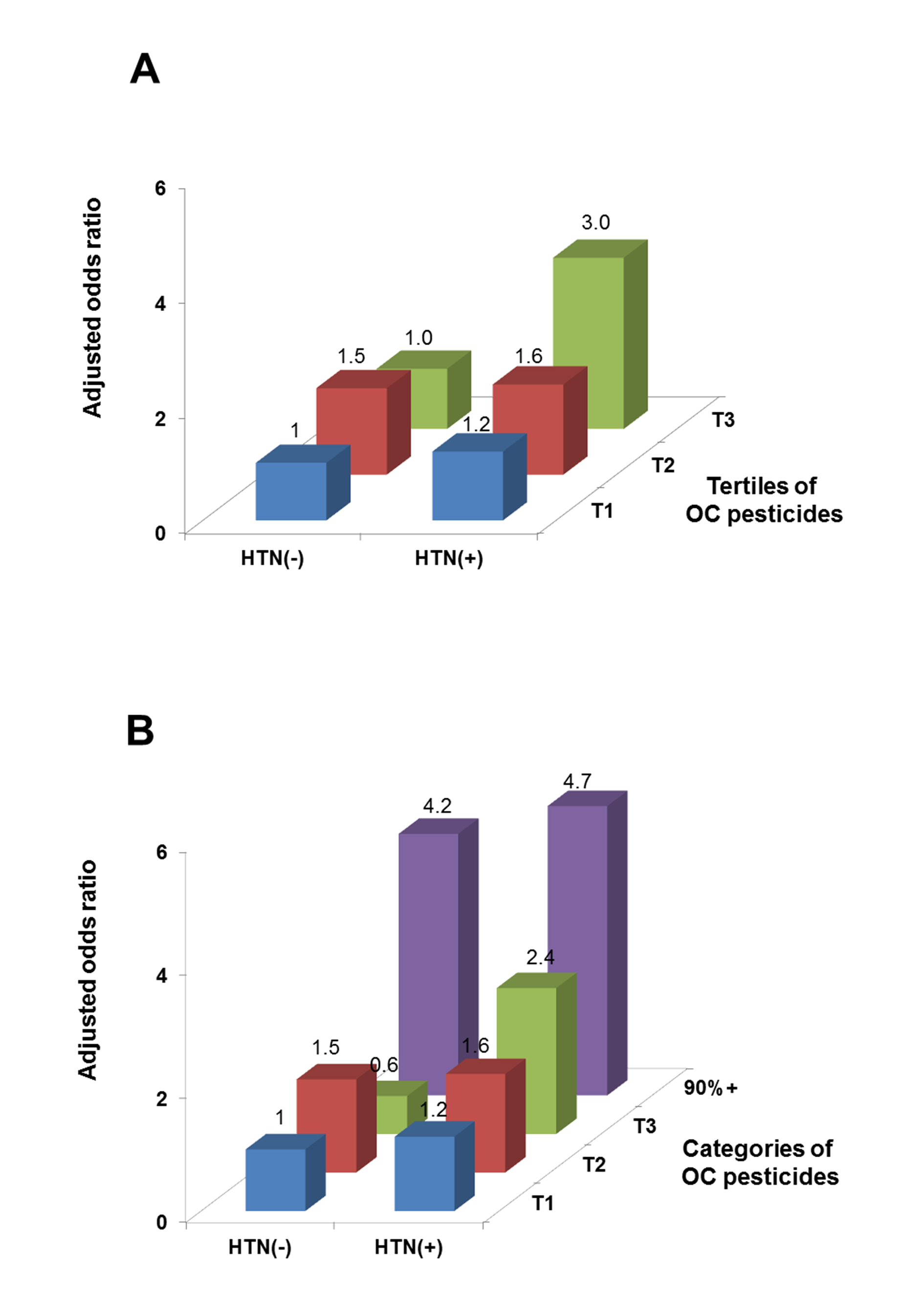


Supplementary figure 1. Associations of hypertension (HTN) and summary measure of organochlorine (OC) pesticides with the risk of low cognitive score. Different from Figure 1, adjusted odds ratios were estimated after considering the design variables and sample weight of National Health and Nutrition Examination Survey (NHANES)
