# Supplementary material for: Can Inconsistent Association between Hypertension and Cognition in Elders be Explained by Levels of Organochlorine Pesticides?
Source: PLoS One. 2015 Dec 2;10(12):e0144205. doi: 10.1371/journal.pone.0144205 (PMC4668046; doi:10.1371/journal.pone.0144205)
Supplement: S1 Table — (DOCX) [file pone.0144205.s002.docx]

Supplementary table 1. Adjusted^*^ means of Digit Symbol Substitution Test score among all subjects or stratified by tertiles of serum concentrations of organochlorine pesticides

|  |  | Hypertension (-)  (n=207) | Hypertension (+)  (n=437) | P _value_ | P_interaction_ |
| --- | --- | --- | --- | --- | --- |
| All subjects |  | 43.1 | 39.8 | <0.01 |  |
| Stratified analyses by tertiles of each compound (median value, ng/g lipid) | | | |  |  |
| p,p’-DDT | T1 (5.7) | 45.7 | 45.1 | 0.77 | 0.08 |
|  | T2 (9.4) | 44.9 | 41.4 | 0.13 |  |
|  | T3 (25.6) | 39.1 | 33.0 | <0.01 |  |
|  |  |  |  |  |  |
| p,p’-DDE | T1 (324.5) | 43.8 | 44.1 | 0.89 | 0.02 |
|  | T2 (940.5) | 44.2 | 40.1 | 0.07 |  |
|  | T3 (2200.0) | 41.9 | 35.1 | <0.01 |  |
|  |  |  |  |  |  |
| β-hexachlorocyclohexane | T1 (12.8) | 44.6 | 42.2 | 0.26 | 0.45 |
|  | T2 (28.5) | 45.0 | 41.1 | 0.07 |  |
|  | T3 (73.4) | 40.3 | 36.0 | 0.07 |  |
|  |  |  |  |  |  |
| Trans-nonachlor | T1 (25.9) | 45.1 | 43.0 | 0.37 | 0.22 |
|  | T2 (48.2) | 45.1 | 41.5 | 0.10 |  |
|  | T3 (88.9) | 39.4 | 34.7 | 0.04 |  |
|  |  |  |  |  |  |
| Oxychlordane | T1 (17.9) | 45.1 | 41.6 | 0.13 | 0.71 |
|  | T2 (31.3) | 45.5 | 42.7 | 0.20 |  |
|  | T3 (54.7) | 38.6 | 35.0 | 0.11 |  |
|  |  |  |  |  |  |
| Heptachlor epoxide | T1 (5.1) | 45.7 | 42.2 | 0.14 | 0.55 |
|  | T2 (10.7) | 44.5 | 40.5 | 0.03 |  |
|  | T3 (23.0) | 37.6 | 36.9 | 0.80 |  |

^*^Adjusted for age, sex, race-ethnicity, education, poverty income ratio, cigarette smoking, body mass index, physician-diagnosed heart diseases, diabetes, and cancer
