# Supplementary material for: Can Inconsistent Association between Hypertension and Cognition in Elders be Explained by Levels of Organochlorine Pesticides?
Source: PLoS One. 2015 Dec 2;10(12):e0144205. doi: 10.1371/journal.pone.0144205 (PMC4668046; doi:10.1371/journal.pone.0144205)
Supplement: S2 Table — (DOCX) [file pone.0144205.s003.docx]

Supplementary table 2. Adjusted^*^ odds ratios (ORs) and 95% confidence intervals (CIs) between hypertension and the risk of low Digit Symbol Substitution Test score (<25^th%^ of study subjects) among all subjects or stratified by serum concentrations of organochlorine (OC) pesticides

|  |  | Hypertension (-)  (n=207) | Hypertension (+)  (n=437) | P _value_ | P_interaction_ |
| --- | --- | --- | --- | --- | --- |
| All subjects | Cases/Subjects at risk | 40/207 | 128/437 |  |  |
|  | Adjusted ORs (95% CIs) | Reference | 1.6 (1.0-2.5) |  |  |
| Stratified analyses by tertiles of each compound | |  |  |  |  |
| p,p’-DDT |  |  |  |  |  |
| T1 | Cases/Subjects at risk | 11/87 | 23/126 |  |  |
|  | Adjusted ORs (95% CIs) | Reference | 1.6 (0.6-4.0) | 0.36 | 0.51 |
| T2 | Cases/Subjects at risk | 12/62 | 40/156 |  |  |
|  | Adjusted ORs (95% CIs) | Reference | 1.1 (0.5-2.7) | 0.84 |  |
| T3 | Cases/Subjects at risk | 17/58 | 65/155 |  |  |
|  | Adjusted ORs (95% CIs) | Reference | 2.5 (1.1-5.8) | 0.03 |  |
| p,p’-DDE |  | Hypertension(-) | Hypertension (+) |  |  |
| T1 | Cases/Subjects at risk | 15/76 | 27/138 |  |  |
|  | Adjusted ORs (95% CIs) | Reference | 0.7 (0.3-1.8) | 0.50 | 0.05 |
| T2 | Cases/Subjects at risk | 12/70 | 38/144 |  |  |
|  | Adjusted ORs (95% CIs) | Reference | 2.1 (0.9-5.0) | 0.10 |  |
| T3 | Cases/Subjects at risk | 13/61 | 63/155 |  |  |
|  | Adjusted ORs (95% CIs) | Reference | 2.8 (1.2-6.9) | 0.02 |  |
| β-hexachlorocyclohexane | | Hypertension(-) | Hypertension (+) |  |  |
| T1 | Cases/Subjects at risk | 18/89 | 27/125 |  |  |
|  | Adjusted ORs (95% CIs) | Reference | 1.3 (0.6-2.8) | 0.54 | 0.24 |
| T2 | Cases/Subjects at risk | 11/64 | 41/151 |  |  |
|  | Adjusted ORs (95% CIs) | Reference | 1.2 (0.5-3.1) | 0.67 |  |
| T3 | Cases/Subjects at risk | 11/54 | 60/161 |  |  |
|  | Adjusted ORs (95% CIs) | Reference | 2.4 (1.0-6.1) | 0.06 |  |
| Trans-nonachlor | | Hypertension(-) | Hypertension (+) |  |  |
| T1 | Cases/Subjects at risk | 14/77 | 34/138 |  |  |
|  | Adjusted ORs (95% CIs) | Reference | 1.1 (0.5-2.6) | 0.85 | 0.08 |
| T2 | Cases/Subjects at risk | 14/67 | 33/147 |  |  |
|  | Adjusted ORs (95% CIs) | Reference | 1.2 (0.5-2.7) | 0.67 |  |
| T3 | Cases/Subjects at risk | 12/63 | 61/152 |  |  |
|  | Adjusted ORs (95% CIs) | Reference | 3.1 (1.2-8.0) | 0.02 |  |
| Oxychlordane | | Hypertension(-) | Hypertension (+) |  |  |
| T1 | Cases/Subjects at risk | 14/83 | 41/130 |  |  |
|  | Adjusted ORs (95% CIs) | Reference | 2.4 (1.0-5.7) | 0.05 | 0.87 |
| T2 | Cases/Subjects at risk | 13/66 | 28/150 |  |  |
|  | Adjusted ORs (95% CIs) | Reference | 0.7 (0.3-1.8) | 0.50 |  |
| T3 | Cases/Subjects at risk | 13/58 | 59/157 |  |  |
|  | Adjusted ORs (95% CIs) | Reference | 2.3 (1.0-5.5) | 0.06 |  |
| Heptachlor epoxide | | Hypertension(-) | Hypertension (+) |  |  |
| T1 | Cases/Subjects at risk | 21/125 | 20/101 |  |  |
|  | Adjusted ORs (95% CIs) | Reference | 1.7 (0.7-3.8) | 0.22 | 0.84 |
| T2 | Cases/Subjects at risk | 10/43 | 34/142 |  |  |
|  | Adjusted ORs (95% CIs) | Reference | 1.6 (0.6-4.1) | 0.34 |  |
| T3 | Cases/Subjects at risk | 15/53 | 58/160 |  |  |
|  | Adjusted ORs (95% CIs) | Reference | 1.2 (0.5-2.9) | 0.64 |  |

^*^Adjusted for age, sex, race-ethnicity, education, poverty income ratio, cigarette smoking, and body mass index.
